# Does experience modulate automatic imitation? A new look

**DOI:** 10.1371/journal.pone.0357821

**Published:** 2026-09-24

**Authors:** Francesca Genovese, Martina Fanghella, Corrado Sinigaglia, Guido Barchiesi

**Affiliations:** 1 Cognition in Action (CIA) Unit, PHILAB, Milano, Italy; 2 Department of Philosophy, Università degli Studi di Milano, Milano, Italy; 3 Department of Philosophy, Stanford University, Stanford, California, United States of America; Northumbria University, UNITED KINGDOM OF GREAT BRITAIN AND NORTHERN IRELAND

## Abstract

Automatic imitation is a stimulus-response compatibility effect wherein observing an action automatically influences motor performance. However, the mechanism underlying this effect remains controversial. Associative Sequence Learning suggests that automatic imitation arises from contingent visual and motor activity associations. Prior studies have shown that exposure to counter-imitative training can alter these visuomotor associations, suggesting that automatic imitation can be modulated by experience. Here, we aim to bring new insight into how this modulation occurs by exploring the time course of automatic imitation before and after counter-imitative training. If automatic imitation is merely a result of contingent associations, as previously suggested, the effect should consistently be modulated following such training. However, our findings show that automatic imitation is not significantly reduced after counter-imitative training at all the tested time points. This suggests that automatic imitation does not depend on contingent association, at least not as currently understood.

## 1. Introduction

Automatic imitation is a stimulus-response compatibility effect, wherein observing another’s action heavily affects the observer’s motor performance. In a typical automatic imitation task, participants are visually presented with one of two actions (task-irrelevant stimuli) and a cue (colors or numbers) indicating the action they have to perform (task-relevant stimuli). Participants usually produce the cue-commanded action faster if the latter is similar to the observed action. Such an effect has been replicated several times since its discovery, employing different experimental variants [1–5].

Automatic imitation has been explained through various mechanisms. The Ideomotor theory posits a tight relationship between perception and action [6–8]. It claims that representing the perceptual consequences of an action is crucial for controlling its execution, as it enables the agent to anticipate the expected sensory feedback when the action is performed correctly. The same mechanism also accounts for automatic imitation: the perception of an event similar to the events one has experienced following one’s own actions facilitates executing that action. A closely related and more general interpretation of the compatibility effects observed in automatic imitation paradigms is offered by frameworks such as Kornblum’s Dimensional Overlap Model [9] and Hommel’s Theory of Event Coding (Theory of Event Coding [10]). According to these accounts, the automatic imitation effect may reflect a general stimulus–response compatibility process arising from representational overlap between the stimulus and response sets. For instance, in Simon-like choice tasks, each alternative response shares more features with one of the observed events than with the other. Consequently, an observed action facilitates the response with which it shares more features and interferes with the alternative response. From this perspective, automatic imitation can be conceived as a particularly strong case of stimulus–response compatibility, owing to the high degree of resemblance between observed and executed action alternatives.

Similarly to the Ideomotor theory, Motor Resonance theory suggests that when observing another’s action, the sensory information concerning that action is automatically transformed into the motor representation involved in planning and executing that action [11,12]. This explains the automatic imitation effect, with the participants performing the observed action faster even if the sensory information concerning that action is task-irrelevant.

The Associative Sequence Learning (ASL) theory differs from the Ideomotor Theory and Motor Resonance by claiming instead that automatic imitation relies on bidirectional sensorimotor contingencies [13–15]. According to ASL, the links between perceptual and action events develop through bidirectional Pavlovian-like associations between sensory and motor experiences. These associations are purely contingent and arise whenever actions happen to be performed and observed together without prioritizing action execution over action observation [14]. The contingent co-occurrence of similar perceptual and action experiences would explain why observing an action might enhance its execution. However, this enhancement would be reduced or disappear if the contingencies differed [16].

In a demonstration that has since become classic, Heyes et al. (2005) [17] provided the first evidence that automatic imitation can be affected by contingent sensorimotor experiences. Two groups of participants underwent two distinct visuomotor training sessions. In the “imitative” training, participants watched a hand extend or flex its fingers and mimicked the observed action. In the “counter-imitative” training, participants were instructed to perform the opposite action to what they saw, flexing their fingers when the hand extended them, and vice versa. Following the training, both groups participated in a single-choice version of an automatic imitation task, extending or flexing their fingers. The training sessions had differing effects on the magnitude of automatic imitation, consistently with ASL predictions. While the imitative group exhibited an automatic imitation effect, the counter-imitative group abolished this effect.

ASL predictions have also been tested at the neurophysiological level through a passive observation paradigm. Catmur et al. (2007) [18] employed transcranial magnetic stimulation (TMS) to stimulate the primary motor cortex and record motor-evoked potentials (MEP) from the first dorsal interosseus (FDI) and abductor digiti minimi (ADM) muscles while participants passively observed index and little finger movements involving those muscles. Before and after imitative training, MEPs in the ADM and FDI muscles were greater when observing little-finger and index-finger movements, respectively. Following counter-imitative training, the MEP amplitude pattern reversed.

Although these findings demonstrated that contingent sensorimotor experiences could modulate automatic imitation, it is still unclear how this modulation occurs. Indeed, two TMS studies on passive action observation produced contrasting results. Barchiesi & Cattaneo (2013) [19] delivered single TMS pulses on the primary motor cortex at four intervals from the onset of a visually presented action (100 ms, 150 ms, 250 ms, 320 ms), showing that counter-imitative training affected visuomotor facilitation only at late time points, leaving earlier ones unaffected. This result was interpreted as the interaction between two mechanisms: a fixed visuomotor transformation responsible for the early motor facilitation effects and a flexible arbitrary visuomotor association mechanism for the later effects. Conversely, Cavallo et al. (2014) [20] found that the counter-imitative training impacted visuomotor facilitation at all time points, where it was found during the pre-training session (200 ms, 250 ms and 320 ms), thus suggesting that the facilitation effect would be due to only one mechanism, which is contingent association.

The present study aims to shed new light on the automatic imitation effect and its potential modulation by describing, on the behavioral front, the high-resolution time course of this effect before and after imitative and counter-imitative training. We leveraged the time course design logic implemented in [19] and [20], employing a double-choice version of the automatic imitation paradigm in which the delay between the task-irrelevant event (the observation of a hand flexing or extending its fingers) and the task-relevant event (a colored cue indicating whether the action to be performed was a flexion or an extension of the fingers) was parametrically modulated.

Importantly, although the temporal dynamics of counter-imitative training have already been investigated using TMS-MEP measures during passive action observation [19,20], no study has examined these predictions at the behavioural level by describing the time course of automatic imitation itself. This distinction is particularly relevant because ASL has been proposed as a common account of the nature of both motor resonance and automatic imitation, two phenomena that are typically measured using substantially different paradigms. Therefore, testing the temporal predictions of ASL in an automatic imitation task provides an important and complementary assessment of the theory.

Our main aim was to assess, for the first time, whether counter-imitative training consistently affects the time course of the automatic imitation effect. In a pre-training session, we tested the presence of automatic imitation across nine delays between the presentation of the colored cue and the observed action. After a counter-imitative training session, we conducted a post-training session similar to the pre-training one. Then, we compared the magnitude of the automatic imitation effect between pre- and post-training sessions, focusing on delays where automatic imitation emerged during the pre-training session.

Suppose automatic imitation entirely depends on sensorimotor contingencies, as assumed by ASL and suggested by Heyes et al. (2005) [17] findings. In that case, one should expect that the counter-imitative training would cause a suppression or (at least) a reduction of the automatic imitation effect in all time points where it emerged in the pre-training session. If this is not the case, and the automatic imitation effect remains unaffected by counter-imitative training at any point during its progression, it would imply that the automatic imitation effect is not, or not only, a matter of contingent association, at least not in the way it has been understood thus far.

## 2. Materials and methods

### 2.1 Sensitivity analysis

Three previous behavioral experiments implementing a “training-test” rationale employed 10 [17], 8 [21] and 12 [22] participants for each training group. To detect even smaller effects resulting from our experimental design, we decided to increase the number of participants for each training group to 40.

The result of a sensitivity analysis performed using MorePower [23] (alpha = 0.05, power = 0.8, 2 trainings x 2 sessions x 9 delays) indicates that employing a total of 80 participants for our experimental design is sufficient for detecting effects as small as Cohen’s f = 0.156 (partial eta-squared = 0.0238), which is typically considered between “small” and “medium” [24].

Data, analysis code, and research materials employed are available at https://osf.io/a3zns/. Data were analyzed using Jamovi [25], JASP [26], R, Version 4.3.2 [27] and the package ggplot2, Version 3.4.4 [28].

### 2.2 Participants

We collected data from 84 participants (right-handed, with no neurological or psychiatric disorders, vision either corrected or normal; recruitment period: 20^th^ March 2023–16^th^ November 2023); four of them did not participate in all the experimental sessions and were excluded from the analyses. Most participants were university students enrolled in courses held at the Department of Philosophy of the University of Milan. The participation provided them with university credits. Two groups of 40 participants were enrolled in the “Counter” experiment (17 women, age: 25.1 + /- 7.60) or in the “Imitative” experiment (17 women, age: 25.2 + /- 5.97). The study complied with the revised Helsinki Declaration (World Medical Association General Assembly, 2008) and was approved by the local Ethical Committee. All participants gave written informed consent for their participation.

### 2.3 Stimuli

Four right-hand pictures on a black background (two male and two female hands) were presented in the experimental sessions. Each hand could be presented with the fingers in three positions: flexed, intermediate (neutrally positioned), or extended. Building on previous research [17,29,30], we leveraged the illusion of movement produced by the rapid transition from images of a neutrally positioned hand to those showing flexed or extended fingers. This solution allows easy control of event timings. Two small, round objects were shown on the images: one placed between the fingers and the other on the dorsal side of the presented hand. They served as the targets of the displayed goal-directed actions. When finger flexion was shown, the action was a goal-directed grasp of the object between the fingers. When finger extension was shown, the action was a goal-directed touch of the external round object with the dorsal side of the fingertips.

### 2.4 Response detection system

#### 2.4.1 Flex-sensor.

As in recent studies [29,31–34], we took advantage of variable resistors to capture participants’ behavior. One key advantage of this method is its capacity to unambiguously identify and record opposing responses along a single continuous dimension, such as the magnitude of an applied force or an effector’s bending amount.

In the present experiment, participants had to produce a flexion or an extension of their right-hand fingers following the appearance of a colored cue on the screen (see the following paragraph). To detect such responses, we mounted a 95 mm bending variable resistor (“flex sensor”) on the index finger of participants’ right hand. During the experiment, participants rested the right-hand area corresponding to the fifth metacarpal bone on a semi-rigid base and positioned their fingers around a foam rubber block for the duration of each session. This setup ensured that, at the onset of each trial, the fingers were midway between extension and flexion, while still allowing fingers’ extension and flexion, thanks to the block softness. See S2 Text in the Supporting information for details.

### 2.5 General experimental structure

Each participant underwent three sessions: a “Pre-training” session (common to both groups), in which a forced double-choice reaction time version of the automatic imitation paradigm was tested. A training session followed the Pre-training: in this session, one group performed a counter-imitative visuomotor training (“Counter” group) while the other performed an imitative visuomotor training (“Imitative” group). Both groups eventually performed a “Post-training” session, similar to the Pre-training session, the following day. Each session lasted approximately 40 minutes.

#### 2.5.1 Automatic imitation time course (pre-training and post-training sessions).

**Fig 1:**
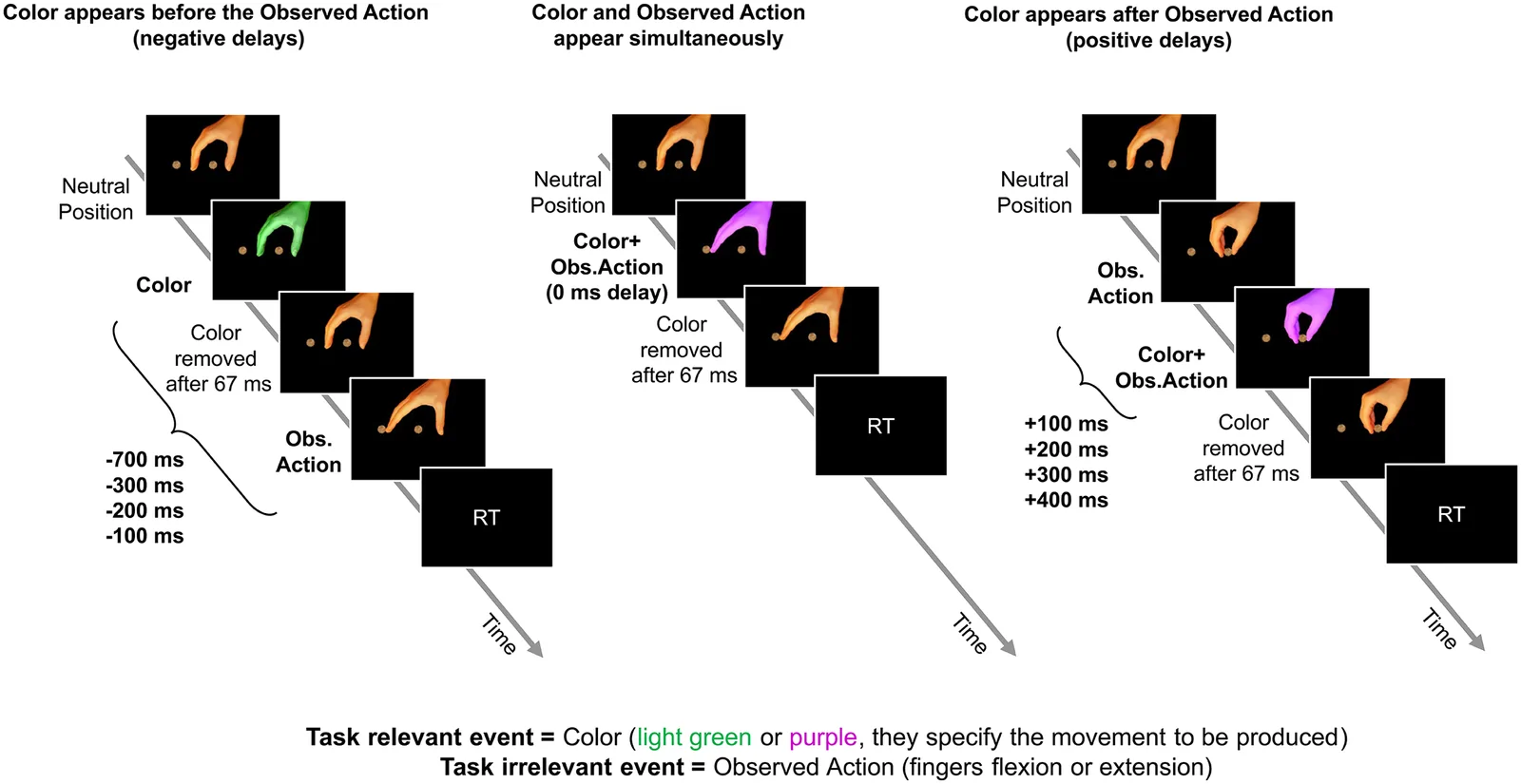
Structure of the automatic imitation task. In each trial of the initial (Pre-training) and the last session (Post-training), the task-relevant event, the colored cue (either light green or purple), appears at one of nine different delays with respect to the task-irrelevant event, the observed action (either fingers flexion or extension). The order of the delays has been randomized across trials. The leftmost timeline depicts a trial where the colored cue flashes on the neutrally positioned hand −700, −300, −200, or −100 ms relative to the subsequent presentation of the observed action (“negative delays trials”). The middle timeline represents a trial where the colored cue and the observed action appear simultaneously (delay = 0 ms). The rightmost timeline represents a trial in which the colored cue appears 100, 200, 300, or 400 ms after the observed action has been presented on the screen (“positive delay trials”). In each trial type, the colored cue lasts 67 ms. The orientation of the displayed hand was randomized across trials between 0 and 360 degrees.

**2.5.1.1 Task and trial structure:** The experimental design was implemented and run by custom Matlab R2021b (Mathworks®) scripts employing Psychtoolbox functions [35–37]. Stimuli were presented on a 60 Hz refresh rate monitor (DELL P2419H, resolution 1920 x 1080 pixels, screen size: 23.8 inches). Each trial presented a randomly oriented (0–360°) neutrally positioned hand on the screen, which lasted randomly between 1000 and 1500 ms. In the remainder of the trial, a colored cue (either light green or purple, lasting 67 ms) and an action (either a flexion or an extension of the fingers, hereafter referred to here as “observed action”) were presented on the screen; the critical feature of each trial was the temporal delay between the presentation of the colored cue and the presentation of the observed action. Here we conceptually grouped them into three categories: in the “negative delays” trials, the neutrally positioned hand turned colored, and after 700, 300, 200, or 100 ms, an action was presented on the screen; in the “simultaneous delay” trials, the colored cue was presented simultaneously with the observed action (0 ms delay); eventually in the “positive delays” trials, the colored cue could be presented 100, 200, 300, 400 ms after the observed action (Fig 1).

The order of the nine delays was randomized across sessions and participants among the whole trial set. The participants’ task was to produce a flexion or an extension of their right-hand fingers according to the presented colored cue as fast as possible and as accurately as possible; the association rule between the color and the movement to be produced was counterbalanced across participants. After either a detected response or the end of the response time window (until 700 ms from the colored cue presentation), participants were provided with a feedback screen for 500 ms: if the response was correct and provided between 150 and 700 ms, their reaction time in milliseconds was displayed; if the detected reaction time was faster than 150 ms, a “Do not guess!” alert was displayed, if it exceeded the deadline time, a “Too Slow” alert was presented; if the provided response was wrong, a “Wrong!” label was shown (feedback labels have been displayed in Italian). Eventually, a black screen was displayed for 1000 ms before the subsequent trial started.

To enhance participants’ motivation and encourage them to perform at their best, we informed them at the beginning of each session that they would receive a prize at the end of the study if they ranked first based on average reaction times and accuracy. Responses beyond the deadline or before the color appeared incurred penalties.

Each Pre-training and Post-training session comprised 720 trials, with 80 trials for each of the nine delays. Within each delay, there were 40 congruent trials, where the observed action matched the movement commanded by the colored cue, and 40 incongruent trials, where the observed actions were the opposite of those commanded by the colored cue. Participants could briefly rest within each session once every 240 trials (2 breaks).

On each trial, at the onset of the colored cue, a TTL trigger was delivered through the parallel port of the presentation PC into a digital input pin of an Arduino board. A custom-made routine, loaded on the board, measured the value of the flex-sensor output and compared it with the flexion and extension thresholds for response detection (see S2 Text in the Supporting information). Once the response and the reaction time were calculated, they were sent back, via serial communication, to Matlab for visual feedback presentation.

Before the Pre-training session, participants underwent a brief familiarization session (~ 5 minutes, 80 trials, see S2 Text in the Supporting information).

#### 2.5.2 Counter-imitative training and imitative training sessions.

The Counter and Imitative groups training sessions comprised 720 trials divided into 10 consecutive blocks. The trial structure was similar to the Pre-training and Post-training sessions but with a few differences: No colored cue was displayed in this session, as the participants’ task was to produce, as quickly and accurately as possible, the opposite action compared to the one they observed in the Counter-Imitiative training, while it was to produce the same action observed in the Imitative training session. Each participant underwent only one of the two trainings, depending on the group they were assigned to. The feedback screens were the same as in the Pre- and Post-training sessions. A further difference compared to the Pre- and Post-training sessions was that the displayed hands were always oriented in an egocentric perspective, in an attempt to mimic a natural visuo-motor contingency acquisition situation (such as self-observation [16],) where the spatial relationship between the effector and the movement sensory outcomes remains stable across time.

### 2.6 Data analysis

#### 2.6.1 Analysis: Pre and post-training sessions.

Only reaction times (RTs) between 150 ms and 700 ms from correct responses were included in the following analyses. To understand whether the automatic imitation effect was differently affected by the two trainings, a mixed three-way 2x9x2 ANOVA was conducted with SESSION (Pre-training and Post-training) and DELAY (−700, −300, −200, −100, 0, 100, 200, 300, and 400 ms) as within-subject factors and TRAINING (Counter and Imitative) as a between-subject factor. To facilitate the interpretation of the results, an index of automatic imitation effect was computed as the dependent variable: we calculated the median RT of the congruent trials, and we subtracted it from the median RT of the incongruent ones separately for each cell of the three-way ANOVA and for each participant. Thus, if participants were slower in the incongruent trials than congruent ones, this would have been reflected as a positive automatic imitation index, and vice versa if incongruent trials were faster than congruent ones.

The analytical strategy followed a hierarchical approach: In the presence of a significant three-way interaction, we planned to conduct follow-up analyses by fitting lower-order ANOVAs to the relevant subsets of the data. These follow-up analyses were performed as independent ANOVA models, with error terms and degrees of freedom estimated within each model, rather than as simple-effects analyses based on the residual variance of the original higher-order ANOVA.

Thus, if a significant three-way interaction was detected, we separated the three-way design into two two-way 2x9 repeated-measures ANOVAs (one for each training group), with SESSION and DELAY as within-subject factors, to understand whether the training changed the automatic imitation effect in the Post-training compared to the Pre-training session.

If a significant two-way interaction was obtained for one or both training groups we first identified, within the Pre-training session, at which delays an automatic imitation effect was detected; subsequently, we tested whether, at these delays, the automatic imitation effect was modulated in the Post-training session.

To this aim, we performed a one-way repeated-measures ANOVA on the Pre-training session of each relevant group with DELAY as the only factor, followed by nine Bonferroni-corrected t-tests (0.05/9) at each delay.

Eventually, using Bonferroni-corrected paired samples t-tests, we tested the modulation of the automatic imitation effect between the Post- and Pre-training sessions at those delays where an automatic imitation effect was detected in the Pre-training session.

As a post-hoc analysis, we tested a residual automatic imitation effect within the Post-training session using nine Bonferroni-corrected post-hoc tests across delays.

#### 2.6.2 Analysis: Counter and imitative training sessions.

To test participants’ performance improvement during the training sessions we conducted, separately for each group, a one-way repeated-measures ANOVA with TRAINING BLOCKS (from the first to the tenth) as the independent variable and the median RT for each block as the dependent variable. RTs between 150 ms and 700 ms from correct responses were included in the analysis. We fitted a linear-mixed regression model to test a linear trend across the blocks. The model predicted the RTs based on training blocks as a fixed effect, accounting for random intercepts and slopes for training blocks across subjects.

## 3. Results

### 3.1 Results: Pre and Post-training sessions

Given the complexity of the factorial design, here we report only the effects directly relevant to our hypotheses. A full description of the effects is provided in the S1 Text of the Supporting information.

The percentage of trials included in the analysis is 89.8% for the Counter group and 88.4% for the Imitative group.

The mixed three-way ANOVA (TRAINING x SESSION x DELAY) produced a significant three-way interaction (F_6.99, 545.01_ = 3.132; *P* = 0.003; partial-η^2^ = 0.039, Greenhouse-Geisser correction was applied for departure from sphericity), indicating that the automatic imitation effect in the Post-training session was affected differently by the two type of training across delays, compared to the Pre-training session (S1 Text of the Supporting information.

The analysis of the two two-way repeated-measures ANOVAs (SESSION x DELAY) for each training group produced a two-way interaction for the Counter group (F_6.17, 240.76_ = 6.55; *P* < 0.001; partial-η^2^ = 0.14, Greenhouse-Geisser corrected), indicating that the automatic imitation effect in the Post-training session was differently modulated across delays with respect to the Pre-training session.

We did not find such an interaction for the Imitative group (F_6.59, 256.99_ = 0.921; *P* = 0.487; partial-η^2^ = 0.023, Greenhouse-Geisser corrected), indicating that the automatic imitation effect in the Post-training session was not modulated across delays with respect to the Pre-training session (S1 Text of the Supporting information). Given the absence of a two-way interaction within the Imitative group, we did not further analyze the modulation of the automatic imitation effect between the Pre- and Post-training sessions within this training group.

The one-way repeated-measures ANOVA conducted on the delays of the Pre-training session of the Counter group produced a significant effect (S1 Text of the Supporting information) (F_5.88, 229.31_ = 32.8, *P* < 0.001; partial-η^2^ = 0.457 Greenhouse-Geisser corrected), indicating that the automatic imitation effect was modulated across delays. Nine Bonferroni-corrected t-tests against zero (0.05/9 = 0.0056, Table 1) detected a positive automatic imitation effect from 0 to 400 ms delays.

**Table 1:** The *Delay* column indicates the time delays between the presentation of the observed action and the colored cue. The *Congruent* column presents the mean RTs, averaged across all participants, for trials where the observed action matched the action to be performed. The *Incongruent* column displays the mean RTs for trials where the observed and performed actions differed. The *t-test statistic*, *degrees of freedom*, *effect size*, and *p-value* columns report the statistical outcomes related to the automatic imitation effect, with significant effects highlighted in bold. A Bonferroni-corrected significance threshold of *P* < 0.0056 was applied to account for the nine comparisons.

Counter Group															
Pre-training								Post-training							
Delay (ms)	Incongruent (ms)	Congruent (ms)	Automatic Imitation (ms)	T-stat	df	Cohen’s d	P-value *(p < 0.0056)*	Delay (ms)	Incongruent (ms)	Congruent (ms)	Automatic Imitation (ms)	T-stat	df	Cohen’s d	P-value *(p < 0.0056)*
−700	384	387	−3	−0.86	39	−0.14	0.393	−700	370	366	4	1.50	39	0.24	0.141
−300	391	384	7	2.12	39	0.34	0.040	−300	366	370	−4	−1.39	39	−0.22	0.173
−200	383	383	0	0.11	39	0.02	0.916	−200	370	366	4	2.19	39	0.35	0.034
−100	389	383	6	1.61	39	0.25	0.116	−100	370	365	5	1.73	39	0.27	0.091
0	396	372	24	6.37	39	1.01	***< .001***	0	373	352	21	7.14	39	1.13	***< .001***
100	394	347	47	12.15	39	1.92	***< .001***	100	366	335	31	7.91	39	1.25	***< .001***
200	383	336	47	11.18	39	1.77	***< .001***	200	352	329	23	6.01	39	0.95	***< .001***
300	375	336	39	8.40	39	1.33	***< .001***	300	341	320	21	5.71	39	0.90	***< .001***
400	367	332	35	8.65	39	1.37	***< .001***	400	339	322	17	3.52	39	0.56	***0.001***
**Imitative Group**															
**Pre-training**								**Post-training**							
**Delay (ms)**	**Incongruent (ms)**	**Congruent (ms)**	**Automatic Imitation (ms)**	**T-stat**	**df**	**Cohen’s d**	**P-value *(p < 0.0056)***	**Delay (ms)**	**Incongruent (ms)**	**Congruent (ms)**	**Automatic Imitation (ms)**	**T-stat**	**df**	**Cohen’s d**	**P-value *(p < 0.0056)***
−700	385	383	2	0.66	39	0.10	0.513	−700	368	371	−3	−0.85	39	−0.14	0.398
−300	385	385	0	0.02	39	0.00	0.985	−300	368	373	−5	−1.79	39	−0.28	0.082
−200	382	382	0	−0.16	39	−0.03	0.871	−200	372	366	6	1.89	39	0.30	0.066
−100	389	378	11	2.95	39	0.47	***0.005***	−100	375	362	13	3.66	39	0.58	***< .001***
0	398	369	29	6.90	39	1.09	***< .001***	0	387	352	35	8.24	39	1.30	***< .001***
100	386	343	43	9.11	39	1.44	***< .001***	100	374	326	48	12.34	39	1.95	***< .001***
200	380	333	47	9.13	39	1.44	***< .001***	200	364	318	46	13.31	39	2.10	***< .001***
300	372	326	46	9.50	39	1.50	***< .001***	300	357	312	45	13.25	39	2.10	***< .001***
400	366	326	40	7.60	39	1.20	***< .001***	400	349	312	37	8.20	39	1.30	***< .001***

We then tested whether the automatic imitation effect was reduced between the Post-training and Pre-training sessions within the Counter group. We limited the testing to those delays in which the automatic imitation was found in the Pre-training session (0, + 100, + 200, + 300, + 400 ms). The paired sample t-tests (Bonferroni corrected for five comparisons) showed that, compared to the Pre-training session, Post-training automatic imitation effects were reduced from +100 ms to 400 ms delays (all Ps < 0.01; see Table 2). In contrast, they were not modulated at the 0 ms delay, i.e., at the first delay in which automatic imitation emerged in the Pre-training session (t_39_ = 0.52, *P* = 0.604, Cohen’s d = 0.083).

**Table 2:** Pre vs. Post automatic imitation effect (Counter group).

Delay (ms)	Pre (ms)	Post (ms)	t-statistic	df	Cohen’s d	P-value *(p < 0.01)*
0	23.238	20.987	0.5236	39	0.08279	0.604
100	46.775	31.188	4.4742	39	0.70744	***< .001***
200	46.938	23.163	4.877	39	0.77112	***< .001***
300	39.312	21.45	3.0773	39	0.48656	***0.004***
400	34.237	16.587	3.7388	39	0.59115	***< .001***

Eventually, as a post-hoc analysis, we tested the (residual) presence of the automatic imitation effect in the Counter group’s Post-training session. As in the Pre-training session, the automatic imitation effect was detected from the 0 ms delay onward (Bonferroni corrected for nine comparisons) (Table 1).

**Fig 2:**
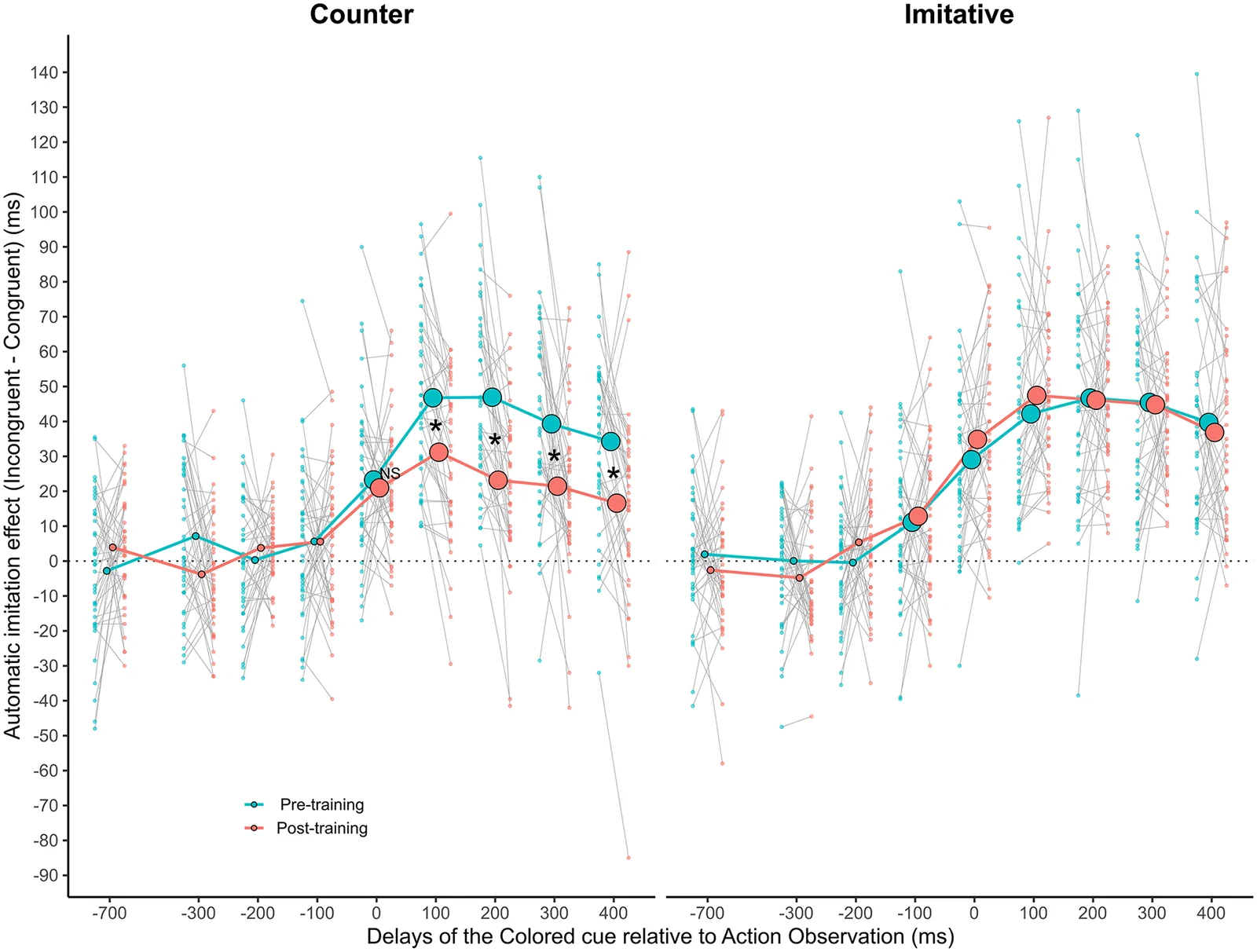
The plot shows the average time course of the automatic imitation effect (RTs from incongruent trials minus RTs from congruent trials) within the Counter group (left) and the Imitative group (right), both in the Pre-training (thick turquoise line) and Post-training session (thick salmon line). Dots on thick lines represent delays at which the automatic imitation effect is significantly greater than zero (Bonferroni corrected for nine comparisons separately for each session and each group). For each delay where a significant automatic imitation was found in the Pre-training session, we compared the magnitude of the automatic imitation effect between Pre-training and Post-training sessions. The NS label indicates a non-significant difference between Pre- and Post-training sessions. In contrast, the “*” symbol indicates a significant difference in automatic imitation magnitude between the two sessions (Bonferroni corrected for five comparisons). No labels or symbols are present in the Imitative group plot since no two-way interaction has been detected; indeed, no statistical testing has been conducted relating the differences between automatic imitation effects between the Pre-training and the Post-training sessions. Thin grey lines at each delay show single-subject data. Within each delay, the left side of each thin line (small turquoise dots) identifies Pre-training data, and the right (small salmon dots) identifies Post-training data.

Although we did not further investigate the Imitative group following the absence of a significant Session × Delay interaction in the two-way ANOVA, we nevertheless performed nine Bonferroni-corrected paired-sample t-tests for each session across delays to facilitate the interpretation of Fig 2 (see Fig 2 and Table 1).

As Bayesian analyses are particularly useful for quantifying the relative evidence for the null and alternative hypotheses [38], we complemented the frequentist analysis with a Bayesian paired-samples t-test to estimate the evidence supporting the absence of a reduction in automatic imitation at the 0-ms delay. The analysis was conducted in JASP [26] using the default Cauchy prior width (r = 0.707). Consistent with our directional prediction, we tested the hypothesis that the automatic imitation effect was reduced in the Post-training compared to the Pre-training session (one-tailed). The resulting Bayes factor was BF + 0 = 0.269 (error < 0.0001), corresponding to BF0+ = 3.72. Thus, the observed data were approximately 3.7 times more likely under the null hypothesis than under the alternative hypothesis. In other words, the data provide moderate evidence in favour of the absence of a reduction in the automatic imitation effect at the 0-ms delay following counter-imitative training.

### 3.2 Results: Counter and Imitative training sessions

**Fig 3:**
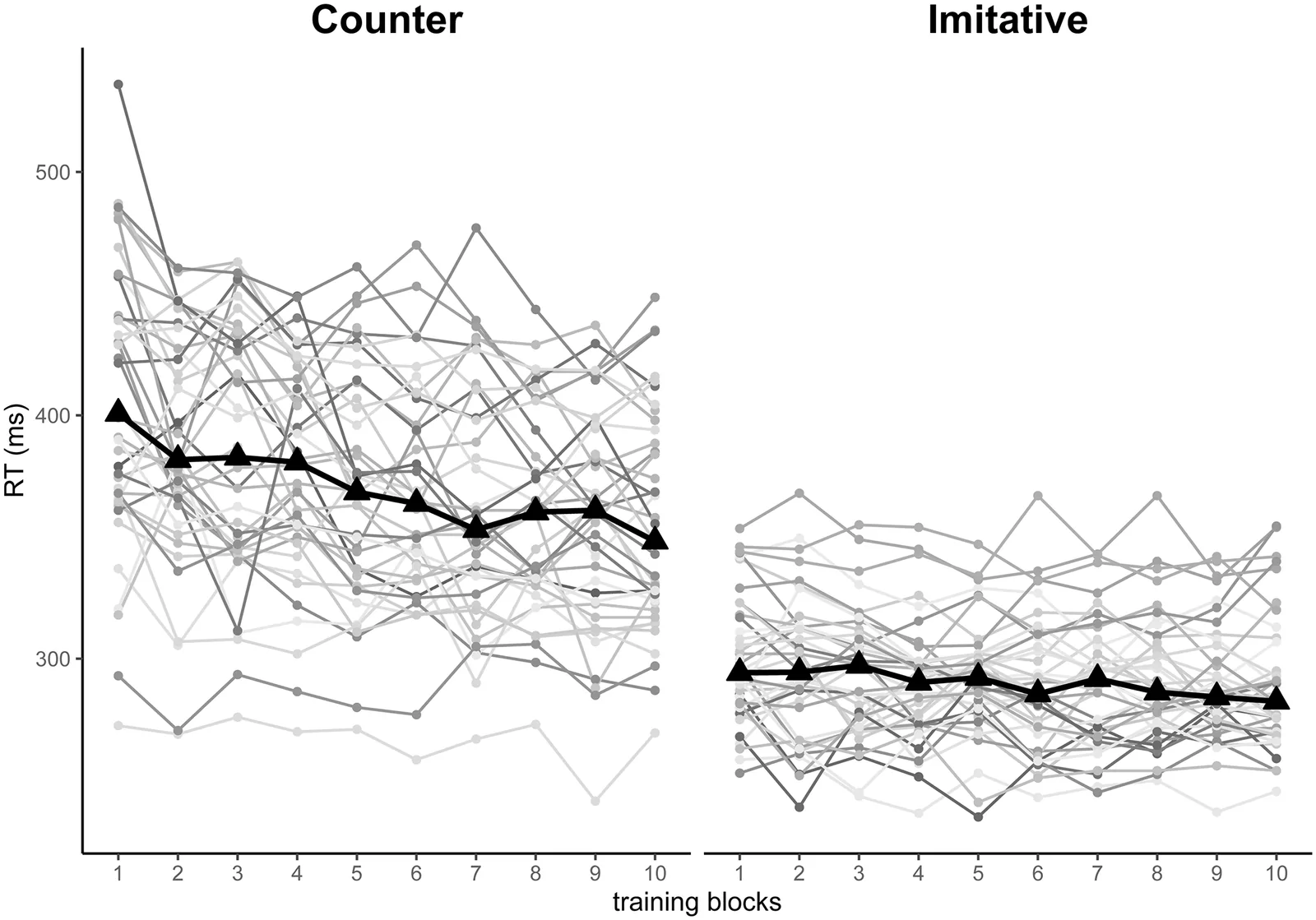
The plot represents the modulation of the RTs across the training blocks for the Counter group (left) and the Imitative group(right). The black triangles connected by the thick black line show the average effect of the training across the ten blocks. The grey dots connected by the thin lines of different shades of grey represent the median RT value of each participant at each training block.

The percentage of trials included in the analysis is 89.5% for the Counter group and 94.2% for the Imitative group. The one-way repeated-measures ANOVA conducted on the training session of the Counter group produced a main effect of the TRAINING BLOCKS variable (F_3.89,151.82_ = 19.26, *P* < 0.001, partial-η^2^ = 0.33, Greenhouse-Geisser corrected). A significant linear trend has been detected across the counter-imitative training blocks, showing an average RT decrease of 5 ms per training block (estimated −5.0037, SE = 0.72, t_39_ = −6.977, P < 0.001). Also, for the Imitative group, the one-way repeated-measures ANOVA produced a main effect of the TRAINING BLOCKS variable (F_5.84, 227.85_ = 3.50, *P* < 0.003, partial-η^2^ = 0.08, Greenhouse-Geisser corrected). A significant linear trend has been detected across imitative training blocks, showing an average RT decrease of 0.97 ms per training block (estimated −0.9729, SE = 0.3137, t_38.99_ = −3.102, *P* < 0.004, Fig 3).

## 4. Discussion

In the present study, we tested the time course of the automatic imitation effect by employing a forced-choice reaction time paradigm in which we parametrically varied the delay between the task-relevant event (a colored cue) and the task-irrelevant event (the observed action) before and after an imitative or a counter-imitative training. This allowed us to investigate whether counter-imitative training abolished or at least reduced the automatic imitation effect. In particular, we aimed to assess whether the putative abolishment or reduction was detected at every delay between the observed action and the colored cue, where automatic imitation was present during the Pre-training session.

Our results showed that the automatic imitation effect is already present when the delay between the colored cue and the action observed is 0 ms and lasts until 400 ms. Strikingly, our results highlighted that the automatic imitation effect was modulated but not abolished after counter-imitative training. We detected a consistent reduction of the automatic imitation effect in the Counter group during the Post-training session. However, this reduction occurred only when the colored cue was presented between 100 and 400 ms after the action observed. No reduction was detected at 0 ms. Importantly, we complemented our results with a Bayesian t-test which supported the claim that automatic imitation within the Post-training session is not reduced at 0 ms delay with respect to the automatic imitation detected in the Pre-training session. For the Imitative group, we did not find any modulatory effect of the imitative training between the Pre- and Post-training sessions. This suggested that the modulation caused by counter-imitative training was training-specific.

These results contrast with those of Heyes et al. (2005) [17], who found a non-significant difference between incompatible and compatible actions after counter-imitative training. To fully evaluate this contrast, it is essential to remember that our and Heyes et al.’s studies differ in some critical features.

Unlike [17], we observed a statistically significant residual automatic imitation effect following counter-imitative training. In the incompatible-training group, Heyes et al. reported a non-significant compatibility effect of 9 ms (p = .194), whereas in our Counter group we observed a significant effect of approximately 21 ms at the 0 ms delay (p < .001). One possible explanation is that our larger sample size increased the sensitivity for the detection of residual effects. However, the residual effects observed in the two studies also differ in magnitude, suggesting that differences in statistical power alone are unlikely to provide a complete explanation for the discrepancy.

A more plausible explanation lies in the procedural differences between the two paradigms. In [17], the observed action served as the imperative stimulus and therefore represented a task-relevant event. In contrast, in our paradigm the observed action was task-irrelevant, and participants responded to a separate colored cue. Furthermore, in Heyes et al., the same observed actions were used throughout both training and testing, whereas in our study the task-relevant stimulus changed across sessions. Consequently, it is possible that the counter-imitative task-set acquired during training was more strongly reinstated during the post-training test in Heyes et al. than in the present study.

Importantly, however, these considerations do not readily follow from the ASL account itself. In both studies, participants underwent highly similar counter-imitative training procedures, which constitute the critical source of contingency-induced remapping according to ASL. Therefore, if counter-imitative experience directly remaps the visuomotor mechanisms responsible for automatic imitation, similar conclusions would be expected regardless of whether the observed action serves as a go-signal or as a task-irrelevant stimulus.

Another possible interpretation of the discrepancy between the findings of Heyes et al. (2005) and those of the present study is that compatibility effects in automatic imitation tasks reflect two contributions: a domain-general stimulus–response compatibility process arising from representational overlap between the stimulus and response sets, which may contribute more strongly in a choice task due to the greater response-selection demands, and a more imitation-specific process linking action observation to the corresponding motor representation. If counter-imitative training primarily weakened the imitation-specific component while leaving the response-selection relatively unaffected, the abolition of the compatibility effect reported by Heyes et al. might reflect the weakening of the former component in a task involving minimal response-selection demands. By contrast, in our choice task, the preserved response-selection component demands produced the post-training residual, but still significant, compatibility effect observed at delays longer than 100 ms.

Although intriguing, this interpretation does not provide a compelling explanation of our findings. First, during counter-imitative training, participants were required not only to suppress the corresponding response but also to repeatedly select and execute the alternative response as quickly as possible. It is therefore unclear why training should selectively modify the imitation-specific component while leaving the contribution associated with the contribution of response-selection processes relatively unaffected. Second, the imitation-specific component should contribute to performance in both single- and two-choice paradigms. If counter-imitative training selectively weakened this component, at least some attenuation of the compatibility effect should also have emerged at the 0 ms delay in the present experiment, consistently with the reduction reported by Heyes et al. (2005). The absence of any such attenuation is difficult to reconcile with this account.

Taken together, we believe that methodological differences between the two paradigms likely contributed to the discrepancy between the findings. However, it remains unclear how these differences could be accommodated within the straightforward ASL prediction that counter-imitative experience remaps the visuomotor mechanisms underlying automatic imitation.

Automatic imitation is a behavioral effect thought to arise due to visuomotor mappings that facilitate the corresponding motor representation connected to the action observed [1–3]. ASL states that these mappings arise from contingent visual and motor activity associations. When contingencies vary, the same associative mechanism producing automatic imitation in the Pre-training session abolishes or reduces this effect in the Post-training session [13–15].

If this were the case, we would have expected to find that, for each delay at which the automatic imitation effect was found in the Pre-training session of the Counter group, a corresponding abolishment or reduction would have also been detected in the Post-training session. However, our results indicate a reduction in the automatic imitation effect is present after a counter-imitative training session, but only at some delays where automatic imitation was found during the Pre-training session.

A dual-route model might explain the differential impact of counter-imitative training on the automatic imitation effect. Dual-route models have been applied to many different domains, ranging from attention [39] to decision-making [40] and moral cognition [41]. Although these models are often characterized in various (and sometimes incompatible) ways [42], they usually share the minimal assumption that the condition that influences whether one process or mechanism occurs diverges from the conditions that influence whether another process or mechanism occurs.

According to the dual-route model, the time course of automatic imitation could result from the interaction of two different visuomotor mechanisms: a rigid and faster visuomotor mapping, similar to that postulated by the Ideomotor and Motor resonance theories, that would cause the automatic imitation effect, and a more flexible and slower “arbitrary association” mapping [19], that the counter-imitative training session would have induced. According to this model, action observation after the counter-imitative training would trigger the automatic imitation route, which facilitates motor responses similar to those observed, and the arbitrary association route, which facilitates the opposed motor responses. While the automatic imitation route is fast, the arbitrary association route takes more time to implement.

Similarly, [43] invoked a dual-route model to account for the disruption of the Simon effect caused by a previous counter-spatial compatibility session. They postulated the interaction between “long-term” and “short-term” memory links. The counter-spatial training did not modify the long-term links, as their effects resulted in the short-term links. The former conceptually corresponds to the rigid visuomotor mapping in our model, while the latter corresponds to our “arbitrary association” mapping.

A second possibility is that the counter-imitative training does not establish a new counter-imitative mapping but simply trains a “reactive” inhibitory process that eventually will be triggered by the specific observed action (observe fingers flexion → inhibit fingers flexion). This is an interesting hypothesis as the inhibitory process itself may require time to develop following action observation, thereby affecting only later stages of the automatic imitation time course. Such an account could potentially explain the temporal selectivity of the observed effect.

Thus, the present data do not allow us to distinguish between a simple counter-imitative visuomotor mapping of an arbitrary-association route and a reactive inhibitory mechanism to the action observed. Indeed, these alternatives are likely not mutually exclusive. Speculating, as already mentioned, we believe that it is unlikely that the counter-training trained only an inhibitory process, because the task involved also a specific action selection to respond with the action opposite to the one observed. However, even in the case of an inhibitory mechanism alone, the results are still difficult to be interpreted as a remapping within a single route mechanism as predicted by ASL, given the time-course observed in the present work.

Instead, the building of a new (counter-imitative) visuomotor mapping during the counter-imitative training may plausibly involve both the activation of an alternative response and the suppression of the automatically activated imitative response. Consistent with many models of action selection [44], the successful selection of one response may depend not only on its activation but also on the inhibition of competing responses, which is likely even more important in the case of facilitated response alternatives.

Therefore, while inhibitory control may contribute to the late modulation observed after counter-imitative training, we speculate that such an inhibition process does not participate alone, but might be part of the arbitrary-association route: the reactive inhibition becomes part of the arbitrary-association route, which has been trained not only to “observe fingers flexion → do fingers extension”, but to “observe fingers flexion → inhibit fingers flexion and do fingers extension.

Although the dual-route model fits our results, a contingency-based explanation for the automatic imitation effect is possible. Indeed, we cannot rule out the possibility that a (significantly) more extended training might also affect the visuomotor mechanism responsible for the early automatic imitation effect. However, the reasons why this should happen are not provided by ASL theory. Furthermore, consider the hypothesis that lifelong Pavlovian-like visuomotor contingencies are responsible for the automatic imitation effect. In that case, these visuomotor links should be expected to resist a relatively brief counter-imitative training. Contrary to what was hypothesized by ASL theory, our results suggest that the brief counter-imitative training employed in our experimental design does not modify such links. Importantly, our results do not imply that experience does not play a role in developing the visuomotor mappings responsible for the automatic imitation effect; rather, they suggest that a brief counter-imitative training is not enough to influence them.

Eventually, it is possible that participants in the Counter group became faster after the training compared to those from the Imitative group; however, we ruled out such an explanation as a supplementary analysis of the absolute RTs did not yield a significant training group x session (see S3 Text of the Supporting information).

Our results contrast with the TMS experiment by [20], who found that counter-imitative training impacted visuomotor facilitation at all time points. Conversely, they align with the TMS study by [19]. A note of caution must be taken when comparing results from MEPs and behavioral experiments. While the logic of the present experimental design has been inspired by [19] and [20], a direct comparison between the present results and the TMS ones may not be so immediate, as they might measure slightly different constructs [45]. For example, when considering the “test-training-test” approach, there is evidence that MEPs amplitude is reversed between effectors after counter-imitative training (see [18]); however, after a similar training, no inversion of the automatic imitation effect is detected (see also [17]). Another important distinction is that the TMS experiments mostly rely on passive observation, while in automatic imitation studies, participants actively prepare actions that will eventually be produced at cue appearance.

Automatic imitation exhibits a specific time course. Exploring this time course allows us to better understand whether and to what extent experience can modulate the automatic imitation effect. We provided evidence that such an effect cannot be reduced to mere sensorimotor contingencies, at least not in the way it has been understood thus far.

## Supporting information

S1 Text Pre- and Post-training results. (DOCX)

S2 Text Apparatus description. (DOCX)

S3 Text Alternative explanation of the results. (DOCX)

## References

[1] Brass M, Bekkering H, Wohlschläger A, Prinz W. Compatibility between observed and executed finger movements: comparing symbolic, spatial, and imitative cues. Brain Cogn. 2000;44(2):124–43. doi: 10.1006/brcg.2000.1225 11041986

[2] Brass M, Bekkering H, Prinz W. Movement observation affects movement execution in a simple response task. Acta Psychol (Amst). 2001;106(1–2):3–22. doi: 10.1016/s0001-6918(00)00024-x 11256338

[3] Stürmer B, Aschersleben G, Prinz W. Correspondence effects with manual gestures and postures: a study of imitation. J Exp Psychol Hum Percept Perform. 2000;26(6):1746–59. doi: 10.1037/0096-1523.26.6.1746 11129371

[4] Cracco E, Bardi L, Desmet C, Genschow O, Rigoni D, De Coster L, et al. Automatic imitation: a meta-analysis. Psychol Bull. 2018;144(5):453–500. doi: 10.1037/bul0000143 29517262

[5] Heyes C. Automatic imitation. Psychol Bull. 2011;137(3):463–83. doi: 10.1037/a0022288 21280938

[6] Greenwald AG. Sensory feedback mechanisms in performance control: with special reference to the ideo-motor mechanism. Psychol Rev. 1970;77(2):73–99. doi: 10.1037/h0028689 5454129

[7] Prinz W. An ideomotor approach to imitation. In: Perspectives on imitation. The MIT Press; 2005. doi: 10.7551/mitpress/5330.003.0006

[8] Neumann O, Prinz W. A common coding approach to perception and action. In: Neumann O, Prinz W, editors. Relationships between perception and action. Springer Berlin Heidelberg; 1990. p. 167–201. doi: 10.1007/978-3-642-75348-0_7

[9] Kornblum S, Hasbroucq T, Osman A. Dimensional overlap: cognitive basis for stimulus-response compatibility--a model and taxonomy. Psychol Rev. 1990;97(2):253–70. doi: 10.1037/0033-295x.97.2.253 2186425

[10] Hommel B, Müsseler J, Aschersleben G, Prinz W. The Theory of Event Coding (TEC): a framework for perception and action planning. Behav Brain Sci. 2001;24(5):849–78; discussion 878-937. doi: 10.1017/s0140525x01000103 12239891

[11] Rizzolatti G, Sinigaglia C. The functional role of the parieto-frontal mirror circuit: interpretations and misinterpretations. Nat Rev Neurosci. 2010;11(4):264–74. doi: 10.1038/nrn2805 20216547

[12] Rizzolatti G, Sinigaglia C. The mirror mechanism: a basic principle of brain function. Nat Rev Neurosci. 2016;17(12):757–65. doi: 10.1038/nrn.2016.135 27761004

[13] Catmur C, Walsh V, Heyes C. Associative sequence learning: the role of experience in the development of imitation and the mirror system. Philos Trans R Soc Lond B Biol Sci. 2009;364(1528):2369–80. doi: 10.1098/rstb.2009.0048 19620108 PMC2865072

[14] Cook R, Bird G, Catmur C, Press C, Heyes C. Mirror neurons: from origin to function. Behav Brain Sci. 2014;37(2):177–92. doi: 10.1017/S0140525X13000903 24775147

[15] Heyes C. Where do mirror neurons come from? Neurosci Biobehav Rev. 2010;34(4):575–83. doi: 10.1016/j.neubiorev.2009.11.007 19914284

[16] Ray E, Heyes C. Imitation in infancy: the wealth of the stimulus: imitation in infancy. Dev Sci. 2011;14(1):92–105. doi: 10.1111/j.1467-7687.2010.00961.x 21159091

[17] Heyes C, Bird G, Johnson H, Haggard P. Experience modulates automatic imitation. Brain Res Cogn Brain Res. 2005;22(2):233–40. doi: 10.1016/j.cogbrainres.2004.09.009 15653296

[18] Catmur C, Walsh V, Heyes C. Sensorimotor learning configures the human mirror system. Curr Biol. 2007;17(17):1527–31. doi: 10.1016/j.cub.2007.08.006 17716898

[19] Barchiesi G, Cattaneo L. Early and late motor responses to action observation. Soc Cogn Affect Neurosci. 2013;8(6):711–9. doi: 10.1093/scan/nss049 22563004 PMC3739914

[20] Cavallo A, Heyes C, Becchio C, Bird G, Catmur C. Timecourse of mirror and counter-mirror effects measured with transcranial magnetic stimulation. Soc Cogn Affect Neurosci. 2014;9(8):1082–8. doi: 10.1093/scan/nst085 23709352 PMC4127010

[21] Press C, Gillmeister H, Heyes C. Sensorimotor experience enhances automatic imitation of robotic action. Proc R Soc B Biol Sci. 2007;274(1625):2509–14. doi: 10.1098/rspb.2007.0774 17698489 PMC2275887

[22] Cook R, Press C, Dickinson A, Heyes C. Acquisition of automatic imitation is sensitive to sensorimotor contingency. J Exp Psychol Hum Percept Perform. 2010;36(4):840–52. doi: 10.1037/a0019256 20695703

[23] Campbell JID, Thompson VA. MorePower 6.0 for ANOVA with relational confidence intervals and Bayesian analysis. Behav Res Methods. 2012;44(4):1255–65. doi: 10.3758/s13428-012-0186-0 22437511

[24] Perugini M, Gallucci M, Costantini G. A practical primer to power analysis for simple experimental designs. Int Rev Soc Psychol. 2018;31(1):20. doi: 10.5334/irsp.181

[25] Jamovi project. jamovi (Version 2.5); 2024 [cited 2024 May 13]. Available from: https://www.jamovi.org/

[26] JASP Team. JASP (Version 0.19.0)[Computer software]; 2024.

[27] R Core Team. R: the R project for statistical computing; 2022 [cited 2024 May 13]. Available from: https://www.r-project.org/

[28] Wickham H. Ggplot2. Springer Science+Business Media, LLC; 2016.

[29] Barchiesi G, Cattaneo L. Motor resonance meets motor performance. Neuropsychologia. 2015;69:93–104. doi: 10.1016/j.neuropsychologia.2015.01.030 25619846

[30] Ubaldi S, Barchiesi G, Cattaneo L. Bottom-up and top-down visuomotor responses to action observation. Cereb Cortex. 2015;25(4):1032–41. doi: 10.1093/cercor/bht295 24132640

[31] Barchiesi G, Zazio A, Marcantoni E, Bulgari M, Barattieri di San Pietro C, Sinigaglia C, et al. Sharing motor plans while acting jointly: a TMS study. Cortex. 2022;151:224–39. doi: 10.1016/j.cortex.2022.03.007 35447381

[32] Zazio A, Barchiesi G, Ferrari C, Marcantoni E, Bortoletto M. M1-P15 as a cortical marker for transcallosal inhibition: a preregistered TMS-EEG study. Front Hum Neurosci. 2022;16:937515. doi: 10.3389/fnhum.2022.937515 36188169 PMC9523880

[33] Cattaneo L, Maule F, Tabarelli D, Brochier T, Barchiesi G. Online repetitive transcranial magnetic stimulation (TMS) to the parietal operculum disrupts haptic memory for grasping. Hum Brain Mapp. 2015;36(11):4262–71. doi: 10.1002/hbm.22915 26248663 PMC6869740

[34] Guidali G, Zazio A, Lucarelli D, Marcantoni E, Stango A, Barchiesi G, et al. Effects of transcranial magnetic stimulation (TMS) current direction and pulse waveform on cortico-cortical connectivity: a registered report TMS-EEG study. Eur J Neurosci. 2023;58(8):3785–809. doi: 10.1111/ejn.16127 37649453

[35] Pelli DG. The VideoToolbox software for visual psychophysics: transforming numbers into movies. Spat Vis. 1997;10(4):437–42. doi: 10.1163/156856897x00366 9176953

[36] Brainard DH. The psychophysics toolbox. Spat Vis. 1997;10(4):433–6. doi: 10.1163/156856897X00357 9176952

[37] Kleiner M, Brainard D, Pelli D, Ingling A, Murray R, Broussard C. What’s new in psychtoolbox-3. Perception. 2007 [cited 2020 Aug 25];36(14):1–16. https://nyuscholars.nyu.edu/en/publications/whats-new-in-psychtoolbox-3

[38] Keysers C, Gazzola V, Wagenmakers E-J. Using Bayes factor hypothesis testing in neuroscience to establish evidence of absence. Nat Neurosci. 2020;23(7):788–99. doi: 10.1038/s41593-020-0660-4 32601411 PMC7610527

[39] Schneider W, Shiffrin RM. Controlled and automatic human information processing: I. Detection, search, and attention. Psychol Rev. 1977;84(1):1–66. doi: 10.1037/0033-295X.84.1.1

[40] Kahneman D, Frederick S. Representativeness revisited: attribute substitution in intuitive judgment. In: Griffin D, Kahneman D, Gilovich T, editors. Heuristics and biases: the psychology of intuitive judgment. Cambridge University Press; 2002. p. 49–81. doi: 10.1017/CBO9780511808098.004

[41] Greene JD. Why are VMPFC patients more utilitarian? A dual-process theory of moral judgment explains. Trends Cogn Sci. 2007;11(8):322–3; author reply 323-4. doi: 10.1016/j.tics.2007.06.004 17625951

[42] Evans JSBT, Stanovich KE. Dual-process theories of higher cognition: advancing the debate. Perspect Psychol Sci. 2013;8(3):223–41. doi: 10.1177/1745691612460685 26172965

[43] Tagliabue M, Zorzi M, Umiltà C, Bassignani F. The role of long-term-memory and short-term-memory links in the Simon effect. J Exp Psychol Hum Percept Perform. 2000;26(2):648–70. doi: 10.1037/0096-1523.26.2.648 10811168

[44] Usher M, McClelland JL. The time course of perceptual choice: the leaky, competing accumulator model. Psychol Rev. 2001;108(3):550–92. doi: 10.1037/0033-295x.108.3.550 11488378

[45] Hétu S, Taschereau-Dumouchel V, Meziane HB, Jackson PL, Mercier C. Behavioral and TMS markers of action observation might reflect distinct neuronal processes. Front Hum Neurosci. 2016;10:458. doi: 10.3389/fnhum.2016.00458 27683548 PMC5021688

