# Supplementary material for: Does experience modulate automatic imitation? A new look
Source: PLoS One. 2026 Sep 24;21(9):e0357821. doi: 10.1371/journal.pone.0357821 (PMC13606974; doi:10.1371/journal.pone.0357821)
Supplement: Supplementary file 1 [file pone.0357821.s001.docx]

***Pre- and Post-Training Results (complete effects report)***

The percentage of trials included in the analysis is 89.8% for the Counter group and 88.4% for the Imitative group. The mixed three-way ANOVA (TRAINING x SESSION x DELAY) produced a significant main effect of SESSION (F1,78 = 10.05, p = 0.002, partial-η2 = 0.114) with the automatic imitation effect greater in the Pre-training session compared to the Post-training session (23.1 ms vs. 19.1 ms) and a significant main effect of DELAY (F6,78 = 94.84, p < .001, partial-η2 = 0.549; -700 = 0.12 ms, -300 = -0.35 ms, -200 = 2.26 ms, -100 = 8.78 ms, 0 = 27.03 ms, 100 = 41.9 ms, 200 = 40.72 ms, 300 = 37.75 ms, 400 = 31.85 ms), with later delays showing greater automatic imitation effects. A main effect of TRAINING was found (F1,78 = 8.03, p < 0.006, Counter = 17.97 ms, Imitative = 24.26 ms) with greater automatic imitation effect in the Imitative compared to the Counter training. Moreover, three significant two-way interactions were found: a SESSION x TRAINING (F1,78 = 13.08, p < 0.001, partial-η2 = 0.144), showing that the difference in automatic imitation effect between Pre- and Post-training in the Counter group was greater compared to the Imitative group (counter Pre-training = 22.3 ms, counter Post-training = 13.6 ms, Imitative Pre-training = 24 ms, Imitative post-training = 24.5 ms). A DELAY x TRAINING (F6, 467.63 =3.09, p = 0.006, partial-η2 = 0.038) interaction was found, indicating greater automatic imitation effect in the Imitative group for later delays (Counter group: -700 = 0.56 ms, -300 = 1.69 ms, -200 = 2.04 ms, -100 = 5.59 ms, 0 = 22.12 ms, 100 = 38.98 ms, 200 = 35.05 ms, 300 = 30.38 ms, 400 = 25.41 ms; Imitative group: -700 = -0.32 ms, -300 = -2.38 ms, -200 = 2.48 ms, -100 = 11.98 ms, 0 = 31.95 ms, 100 = 44.82 ms, 200 = 46.4 ms, 300 = 45.11 ms, 400 = 38.28 ms). A SESSION x DELAY interaction (F6.99 = 4.02, p < 0.001, partial-η2 = 0.049) indicated that a greater automatic imitation effect was detected in the Pre-training session for later delays compared to Post-training automatic imitation effect (Pre-training: -700 = -0.43 ms, -300 = 3.60 ms, -200 = -0.07 ms, -100 = 8.37 ms, 0 = 26.14 ms, 100 = 44.47 ms, 200 = 46.82 ms, 300 = 42.37 ms, 400 = 36.97 ms; post: -700 = 0.67 ms, -300 = -4.3 ms, -200 = 4.58 ms, -100 = 9.2 ms, 0 = 27.92 ms, 100 = 39.32 ms, 200 = 34.63 ms, 300 = 33.12 ms, 400 = 26.72 ms). Eventually, a mixed three-way ANOVA (TRAINING x SESSION x DELAY) produced a significant three-way interaction (F6.99 545.01 = 3.132; p = 0.003; partial-η2 = 0.039, indicating that the automatic imitation effect in the Post-training session was affected differently by the two types of training across delays, compared to the Pre-training session. Greenhouse-Geisser correction was applied for departure from sphericity when needed (S1 Table). Following the detection of a three-way interaction in the mixed three-way ANOVA, we explored each group separately to test the effect of each training through two two-way ANOVAs. The Counter group two-way ANOVA produced a main effect of SESSION (F1, 39 = 22.29 p = < 0.001, partial-η2 = 0.364) with greater Pre-training automatic imitation compared to the Post-training and a main effect of DELAY (F5.71, 222.57 =36.02, p < .001, partial-η2 = 0.48) with greater automatic imitation for later delays. A SESSION x DELAY interaction for the Counter group was found (F6.17, 240.76 = 6.55; p < 0.001; partial-η2 = 0.14), indicating that the automatic imitation effect in the Post-training session was differently modulated across delays compared to the Pre-training session. The analysis of the two two-way repeated-measures ANOVAs for the Imitative group produced a significant main effect of DELAY (F5.26, 205.12 = 60.936, p < .001, partial-η2 = 0.61) with greater automatic imitation for later delays. However, we did not find such a two-way interaction for the DELAY x SESSION effect in the Imitative group (F6.59, 256.99 = 0.921; p = 0.487; partial-η2 = 0.023), indicating that the automatic imitation effect in the Post-training session was not modulated across delays with respect to the Pre-training session. (S2 Table).

The one-way repeated-measures ANOVA conducted on the delays of the Pre-training session of the Counter group produced a significant main effect of DELAY (S3 Table) (F5.88, 229.31 = 32.8, p < 0.001; partial-η2 = 0.457 Greenhouse-Geisser corrected), indicating that the automatic imitation effect was increased with greater delays. Nine Bonferroni-corrected t-tests against zero (0.05/9 = 0.0056, Table 1 Main Manuscript) detected a positive automatic imitation effect from 0 to 400 ms delays.

We then tested whether the automatic imitation effect was reduced between the Post-training and Pre-training sessions within the Counter group. We limited the testing to those delays in which the automatic imitation was found in the Pre-training session (0, +100, +200, +300, +400 ms). The paired sample t-tests (Bonferroni corrected for five comparisons) showed that, compared to the Pre-training session, Post-training automatic imitation effects were reduced from +100 ms to 400 ms delays (all ps <0.01; see Table 2 Main Manuscript for full results). In contrast, they were not modulated at the 0 ms delay, i.e., at the first delay in which automatic imitation emerged in the Pre-training session (t39= 0.52 p = 0.604, Cohen’s d = 0.083).

Eventually, as a post-hoc analysis, we tested the (residual) presence of the automatic imitation effect in the Counter group's Post-training session. As in the Pre-training session, the automatic imitation effect was detected from the 0 ms delay onward (Bonferroni corrected for nine comparisons) (Table 1 Main Manuscript).

We did not analyze the Imitative group's interactions further, given the absence of a two-way interaction within the two-way ANOVA. As stated in the Analysis paragraph, we performed nine Bonferroni-corrected t-tests separately for each session across all delays also for the Imitative group.

**S1 Table**

| **Three-way Mixed Anova** | | | | | | |
| --- | --- | --- | --- | --- | --- | --- |
|  |  |  |  |  |  |  |
|  | **Sum of Squares** | **df** | **Mean Square** | **F** | ***p*** | **η²p** |
| **Session** | 5887 | 1 | 5887 | 10.05 | **0.002** | 0.114 |
| **Session ✻ training** | 7666 | 1 | 7666 | 13.08 | **< .001** | 0.144 |
| Residual | 45702 | 78 | 586 |  |  |  |
| **Delay** | 424390 | 6 | 70788 | 94.84 | **< .001** | 0.549 |
| **Delay ✻ training** | 13833 | 6 | 2307 | 3.09 | **0.006** | 0.038 |
| Residual | 349023 | 467.63 | 746 |  |  |  |
| **Session ✻ Delay** | 12319 | 6.99 | 1763 | 4.02 | **< .001** | 0.049 |
| **Session ✻ Delay ✻ training** | 9593 | 6.99 | 1373 | 3.13 | **0.003** | 0.039 |
| Residual | 238901 | 545.01 | 438 |  |  |  |
| **Training** | 14191 | 1 | 14191 | 8.03 | **0.006** | 0.093 |
| Residuals | 137903 | 78 | 1768 |  |  |  |

**S2 Table**

| **Two-way ANOVAs** | | | | | | | | | | | | | |
| --- | --- | --- | --- | --- | --- | --- | --- | --- | --- | --- | --- | --- | --- |
|  |  |  |  |  |  |  |  |  |  |  |  |  |  |
| **Counter Group** | | | | | | | **Imitative Group** | | | | | | |
|  |  |  |  |  |  |  |  |  |  |  |  |  |  |
|  | **Sum of squares** | **df** | **Mean square** | **F** | ***p*** | **η²p** |  | **Sum of squares** | **df** | **Mean square** | **F** | ***p*** | **η²p** |
| **Session** | 13494 | 1 | 13494 | 22.29 | **< .001** | 0.364 | **Session** | 58.7 | 1 | 58.7 | 0.104 | 0.749 | 0.003 |
| Residual | 23611 | 39 | 605 |  |  |  | Residual | 22090.7 | 39 | 566.4 |  |  |  |
| **Delay** | 154813 | 5.71 | 19352 | 36.02 | **< .001** | 0.48 | **Delay** | 283410 | 5.26 | 53885 | 60.936 | **< .001** | 0.61 |
| Residual | 167637 | 222.57 | 537 |  |  |  | Residual | 181386.3 | 205.12 | 884.3 |  |  |  |
| **Session ✻ Delay** | 18931 | 6.17 | 2366 | 6.55 | **< .001** | 0.144 | **Session ✻ Delay** | 2980.6 | 6.59 | 452.3 | 0.921 | 0.487 | 0.023 |
| Residual | 112697 | 240.76 | 361 |  |  |  | Residual | 126204 | 256.99 | 491.1 |  |  |  |

**S3 Table**

| **One-way ANOVA** | | | | | | |
| --- | --- | --- | --- | --- | --- | --- |
|  |  |  |  |  |  |  |
|  | **Sum of Squares** | **df** | **Mean Square** | **F** | **p** | **η²p** |
| **Delay** | 130427 | 5.88 | 22183 | 32.8 | **< .001** | 0.457 |
| Residual | 155240 | 229.31 | 677 |  |  |  |
