# Supplementary material for: Does experience modulate automatic imitation? A new look
Source: PLoS One. 2026 Sep 24;21(9):e0357821. doi: 10.1371/journal.pone.0357821 (PMC13606974; doi:10.1371/journal.pone.0357821)
Supplement: Supplementary file 2 [file pone.0357821.s002.docx]

***Apparatus description***

***1.Flex-sensor***

To record finger movements, we used bending variable resistors (“flex sensors”), which can precisely capture opposing responses along a single continuous dimension, such as the degree of bending. In our task, participants flexed or extended the fingers of their right hand in response to a colored cue on the screen.

A 95 mm flex-sensor was mounted on the dorsal side of the right-hand index finger. Two soft sheaths (heat-shrink sleeves, ~0.5 cm diameter × ~2.5 cm length) were attached to the proximal and middle phalanges and secured with skin adhesive tape (without flattening them). The flex sensor was then inserted into the sheaths and fixed to the index metacarpus. This arrangement allowed the sensor to bend and slide within the sheaths during finger movements, and to return to its original position when the fingers relaxed (Figures S1–S3).

The flex-sensor was connected in series with a fixed resistor in a voltage-divider circuit, powered and read out by an Arduino® board. Voltage variations caused by finger bending were converted into conductivity values. For each session, maximal flexion and extension were measured and mapped to 0 and 1000 arbitrary units, respectively. Response thresholds were set to ±100 units relative to the initial conductivity at cue onset. For example, an initial reading of 430 units yielded extension and flexion thresholds of 530 and 330 units, respectively. During each session, the right hand was concealed under an opaque box to prevent visual feedback.

***2.Foam Rubber Manipulandum***
Between the participant and the PC monitor, a foam rubber column (diagonal ~4.5 cm, height ~14 cm) and a semi-rigid base were fixed to the table. The fifth metacarpal of the right hand rested on the base, with the fingers placed around the foam column. This setup positioned the fingers midway between extension and flexion at trial onset and allowed comfortable flexion due to the column’s softness (Figures S1–S3).

**S1 Fig.** **Example of a participant's hand starting position at the beginning of each trial.** The foam rubber structure (dark grey) keeps the fingers in an intermediate position while waiting for the stimuli to appear on the screen. The hand lays on a semi-rigid base (white), which allows the fingers to move freely during extension by creating space between the table and the fifth metacarpal side of the hand. Additionally, if necessary, a soft foam rubber support has been placed beneath the forearm to maintain alignment of the hand-forearm complex.

**S2 Fig.** Example of fingers extension movement.

**S3 Fig.** Example of fingers flexion movement.

***3. Familiarization Session***

Participants underwent a familiarization session to learn the association between the movements to be produced and the colored cues. The task employed was identical to the one employed in the Pre-training and Post-training sessions, with the difference that the hands on the screen did not move (they only changed color) and that the time window for response production was increased to 3000 ms. Participants underwent the familiarization session only before the Pre-training session.
