# Supplementary material for: Does experience modulate automatic imitation? A new look
Source: PLoS One. 2026 Sep 24;21(9):e0357821. doi: 10.1371/journal.pone.0357821 (PMC13606974; doi:10.1371/journal.pone.0357821)
Supplement: Supplementary file 3 [file pone.0357821.s003.docx]

***Alternative explanation of the results***

Participants in the Counter group (compared to the Imitative group) may have become faster at responding to the imperative cue in the Post-training session, without any genuine modification of the visuomotor mappings targeted by counter-imitative training. In this case, the reduced automatic imitation effect could reflect a general speeding of responses rather than a change in action-observation processes: this response speeding could indeed reduce the time available for the observed action to influence performance

Thus, if participants in the Counter group were simply responding faster in the Post-training session, one would expect systematic changes in absolute RTs across training and sessions, as a faster response would reduce the time available for the observed action to influence performance.

To directly assess this possibility, we conducted additional analyses on the absolute RTs. We thus conducted a four-way ANOVA with training group as between-subject and session, delay, congruence as within-subjects variable with the absolute RTs as dependent variable (not on the automatic imitation effect directly as in the main analysis) with the aim of testing the training x session interaction, which results in F = 1.43, p = 0.23 (see S4 Table). This result does not point to increased speed as the main factor reducing the automatic imitation effect as the general response speed of the two groups does not vary differently across sessions.

**S4 Table**

| Effect | df | MSE | F | pes | p.value |
| --- | --- | --- | --- | --- | --- |
| training | 1, 78 | 49978.08 | 0.01 | <.001 | .943 |
| session | 1, 78 | 3088.46 | 67.54 *** | .464 | <.001 |
| training:session | 1, 78 | 3088.46 | 1.44 | .018 | .234 |
| congruency | 1, 78 | 883.99 | 363.25 *** | .823 | <.001 |
| training:congruence | 1, 78 | 883.99 | 8.03 ** | .093 | .006 |
| delay | 4.03, 314.24 | 697.97 | 237.06 *** | .752 | <.001 |
| training:delay | 4.03, 314.24 | 697.97 | 0.72 | .009 | .581 |
| session:congruence | 1, 78 | 292.96 | 10.05 ** | .114 | .002 |
| training:session:congruence | 1, 78 | 292.96 | 13.08 *** | .144 | <.001 |
| session:delay | 6.16, 480.57 | 268.72 | 0.88 | .011 | .511 |
| training:session:delay | 6.16, 480.57 | 268.72 | 0.76 | .010 | .605 |
| congruence:delay | 6.00, 467.63 | 373.18 | 94.84 *** | .549 | <.001 |
| training:congruence:delay | 6.00, 467.63 | 373.18 | 3.09 ** | .038 | .006 |
| session:congruence:delay | 6.99, 545.01 | 219.17 | 4.02 *** | .049 | <.001 |
| training:session:congruence:delay | 6.99, 545.01 | 219.17 | 3.13 ** | .039 | .003 |

Taken together, these additional analyses do not support the interpretation that the observed modulation of automatic imitation resulted from a general speeding of responses in the Counter group.
